# A powerful latent variable method for detecting and characterizing gene-based gene-gene interaction on multiple quantitative traits

**DOI:** 10.1186/1471-2156-14-89

**Published:** 2013-09-23

**Authors:** Fangyu Li, Jinghua Zhao, Zhongshang Yuan, Xiaoshuai Zhang, Jiadong Ji, Fuzhong Xue

**Affiliations:** 1 Department of Epidemiology and Biostatistics, School of Public Health, Shandong University, Jinan 250012, China; 2 MRC Epidemiology Unit& Institute of Metabolic Science, Addenbrooke’s Hospital, Cambridge CB20QQ, UK

**Keywords:** Thinking quantitatively for complex diseases, Gene-based gene-gene interaction, Quantitative traits, mPLSPM statistic

## Abstract

**Background:**

On thinking quantitatively of complex diseases, there are at least three statistical strategies for analyzing the gene-gene interaction: SNP by SNP interaction on single trait, gene-gene (each can involve multiple SNPs) interaction on single trait and gene-gene interaction on multiple traits. The third one is the most general in dissecting the genetic mechanism underlying complex diseases underpinning multiple quantitative traits. In this paper, we developed a novel statistic for this strategy through modifying the Partial Least Squares Path Modeling (PLSPM), called mPLSPM statistic.

**Results:**

Simulation studies indicated that mPLSPM statistic was powerful and outperformed the principal component analysis (PCA) based linear regression method. Application to real data in the EPIC-Norfolk GWAS sub-cohort showed suggestive interaction (*γ*) between *TMEM18* gene and *BDNF* gene on two composite body shape scores (*γ* = 0.047 and *γ* = 0.058, with *P* = 0.021, *P* = 0.005), and BMI (*γ* = 0.043, *P* = 0.034). This suggested these scores (synthetically latent traits) were more suitable to capture the obesity related genetic interaction effect between genes compared to single trait.

**Conclusions:**

The proposed novel mPLSPM statistic is a valid and powerful gene-based method for detecting gene-gene interaction on multiple quantitative phenotypes.

## Background

In search of novel loci influencing complex traits in humans, successes in genome-wide association studies (GWAS) have been well-documented [1]. While these have greatly improved our understanding of the genetic architecture of complex traits, often implicating biological pathways previously went undetected, most genetic components for complex traits are still to be revealed. One can attribute this to the sub-optimality of their study designs, but inappropriate statistical data analysis strategy, including methods for gene-gene interaction analysis, may also play a role.

Although discussed extensively in the literature, a notable issue remains in GWAS using case–control design [2,3]. Given phenotypes of most complex diseases (obesity, hypertension, diabetes, to name a few) are actually quantitative [4], a case–control design is usually furnished by dividing particular continuous quantitative measurement into case and control groups with a cut off which might not relate so well with genetic variation. Assigning cutoff to a continuous variable can lead to loss of information, and decrease the statistical power caused by selection bias. A proposal revived recently is to treat common disorders as quantitative traits in a framework of thinking quantitatively such that GWAS should be conducted using a population cohort with multiple quantitative traits [4]. In this framework, a complex disease is caused by multiple genes with small effect and their interaction, as well as their interaction with multiple environmental factors. The quantitative phenotype (trait) is expected to be continuous and normally distributed [4-6]. While for some diseases such as body mass index (BMI, weight (in kilograms)/height (in meters)^2^) for obesity, blood pressure for hypertension, and mood for depression the relevant quantitative traits seem obvious, the relevant quantitative traits may not be entirely clear for diseases such as arthritis, autism, cancers, dementia and heart disease for which limited biomarkers are available. Even with obesity, BMI is only a proxy since it crudely measures the mean weight under given body surface area and varies with the amount of body fat and not a representation of its distribution. Various studies have shown that people with abdominal fat (with more weight around the waist) face more risks of cardiovascular diseases [7,8] and other related diseases (such as hypertension, type 2 diabetes, and high cholesterol) [9-11] than those with hip obesity (with more weight around the hip) [10], suggesting that the phenotype of obesity might be more appropriately a synthetically latent trait (SLT) combined from disease-related manifest variables (BMI, waist circumference, hip circumference and neck circumference etc.). This serves as a contrast with most GWASs either using case–control designs [2,3] or using quantitative variables [12-15] with simple linear regression and single SNP-SNP interaction.

To detect gene-gene interaction, at least three statistical strategies can be considered for quantitative phenotypes, including single SNP-SNP interaction on single trait, gene-gene (with multiple SNPs) interaction on single trait and gene-gene (with multiple SNPs) interaction on multiple traits. The first strategy is most susceptible to high false positive rate and low power in detecting modest effects owing to the ignorance of the linkage disequilibrium (LD) information between SNPs [16,17]. Moreover, genes are the functional units in living organisms, analysis by focusing on a gene as a system could potentially yield more biologically meaningful results. In view of this, LD information is used in the second strategy, and some methods aimed at gene-based gene-gene interaction detection exist [18-22]. Based on a gene-based association test –ATOM by combining optimally weighted markers within a gene [18], He et al. extend it to analysis gene–gene interactions [19]. First, they derive the optimal weight for both quantitative and binary traits based on pair-wised LD information and use the principal components (PCs) to summarize the information in each gene. Then, test for interactions between the PCs. In the work of Li and Cui, they conceptually propose a gene-centric framework for genome-wide gene–gene interaction detection [20]. They treat each gene as a testing unit and derive a model-based kernel machine method for two-dimensional genome-wide scanning of gene–gene interactions. Recently, Ma et al. combine marker-based interaction tests between all pairs of markers in two genes to produce a gene-level test for interaction between the two, to test the gene-based gene–gene interaction [21]. The tests are based on an analytic formula derived for the correlation between marker-based interaction tests due to LD. Although, aforementioned methods are proposed to detect the gene-based gene-gene interaction, they fall short of consideration on multiple traits or SLT, especially when the traits are genetic related. It is, therefore, desirable to develop new method to detect gene-gene (with multiple SNPs) interaction on multiple traits.

In this paper, we attempted to develop a novel model for detecting the effect of gene-gene interaction on the SLT summarized by multiple manifest traits. The proposed model was constructed by adding a product term of combined multiple SNPs effect within two genes (genes A and B) via Partial Least Squares Path Modeling (PLSPM) [23,24]. Thus, a structural equation model (SEM) was built between two genes and multiple manifest traits linked by the latent variables of gene A, gene B, gene A × gene B, and multiple traits, so that the gene-gene interaction statistic was defined based on the path coefficient between the latent variables of gene A × gene B and multiple traits. As the path coefficient in proposed statistic was calculated by modifying the Lohmöller PLSPM algorithm [25], we called it the modified PLSPM (mPLSPM) based statistic. Simulation studies were conducted to evaluate its type I error rate and power, and to compare its performance with the PCA-based linear regression model [26-28]. The method was also applied to a real data to evaluate its utility.

## Methods

### Statistical model

Our model is motivated from the original PLSPM which developed from structural equation models (SEM). SEM are complex models allowing the study of real world complexity by taking into account a whole number of causal relationships among latent concepts (i.e. the latent variables (LVs)), each measured by several observed indicators usually defined as manifest variables (MVs). Each path-modeling-based statistic is formed by 2 sub-models: structural (Inner) model and measurement (Outer) model. The structural model indicates the relationships among the latent variables, both of which are inferred from the observed SNPs (from different genes) and traits (e.g. waist, hip, BMI) respectively in this study. The measurement model formulation depends on the direction of the relationships between the latent variables and the corresponding manifest variables. As a matter of fact, different types of measurement model are available: the *reflective model* (or outwards directed model), the *formative model* (or inwards directed model) and the *MIMIC model* (a mixture of the two previous models). The *reflective model* has causal relationships from the latent variable to the manifest variables in its block. In contrast to *reflective* (or effects) model, the *formative* (causal) model has causal relationships from the manifest variables to the latent variables, namely the LV is caused (formed) by the MVs. Its construction is combination of observed (manifest) variables with multidimensional form and aims at minimizing residuals in structural relationships to explain the unobserved (latent) variable with higher *R*^*2*^[23]. More detailed interpretation for the original PLSPM see Additional file 1.

**Figure 1:**
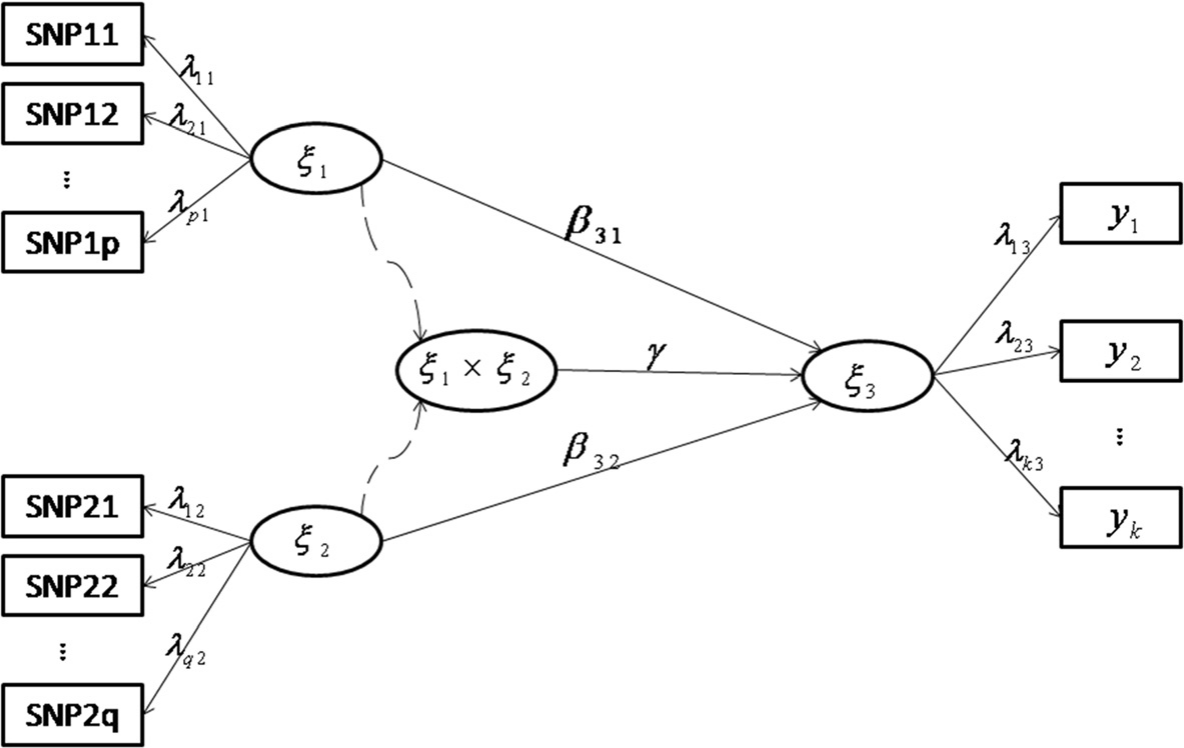
The modified PLSPM-based gene-gene interaction model.

Figure 1 illustrates the framework for our mPLSPM statistic. Let *X*_1_ = (*x*_11_, *x*_12_, …, *x*_1*p*_) and *X*_2_ = (*x*_21_, *x*_22_, …, *x*_2*q*_) denote the genotypes of p SNPs within gene A and q SNPs within gene B, respectively, and *Y* = (*y*_1_, *y*_2_, …, *y*_*k*_) the multiple quantitative measures underlying specific disease, such as the waist circumference, hip circumference and BMI for measuring the human body shape. In this model, latent variables *ξ*_1_ and *ξ*_2_ from the two genes can be derived as with *ξ*_3_ from the quantitative traits. A product term *ξ*_1_ × *ξ*_2_ added to the PLSPM is used to measure the interaction between gene A and gene B, then we can get the structure model: *ξ*_3_ = *β*_0_ + *β*_31_*ξ*_1_ + *β*_32_*ξ*_2_ + *γξ*_1_*ξ*_2_ + *ϵ*. Moreover, path coefficients *β*31, *β*32, and *γ* are the main and interaction effects of gene A and gene B on the phenotype score or SLT (ξ_3_) respectively, while loadings (*λ*′*s*) quantify the relationship between manifest variables (MVs) and their latent variables (LVs). Parameters in the model can be estimated with Lohmöller’s algorithm [23,25], which include the latent variable scores (genetic scores *ξ*_1_, *ξ*_2_, and phenotype score *ξ*_3_), path coefficients (*β*_31_, *β*_32_, and *γ*) and loadings (*λ*′*s*). Specifically, latent variable scores are estimated using linear combinations of their MVs, obtained by an iterative algorithm based on simple/multiple least squares regressions. The path coefficients are derived by regression between dependent LV (*ξ*_3_) and independent LVs (including *ξ*_1_, *ξ*_2_ and their product term *ξ*_1_ × *ξ*_2_) obtained by least squares regression or partial least squares regressions (with higher multicollinearity between independent LVs). Loadings are gotten using regressions of each block of MVs with its LV, obtained by least squares regressions. Since the aim of mPLSPM statistic is mainly to capture the association between effect of SNPs set (genome region) and effect of traits (body shape), and after using “*Cronbach’s alpha*” tool for checking [24], the blocks meet homogeneity and unidimensionality. Therefore the *reflective* model is used to set up the measurement model. At the same time, the impact of multicollinearity between manifests can be alleviated.

In this paper, we modify the Lohmöller’s PLSPM algorithm to estimate the parameters. In details, the specific modified procedure is as follows: 1) working on standardized manifest variables and giving initial values on weights *w*_*ij*_, iteratively alternating the outer and inner estimation steps; 2) specifically in the outer estimation step, the values of the latent variables *ξ*_1_, *ξ*_2_, and *ξ*_3_ were estimated by $\nu_{1}=\sum_{j=1}^{p}\omega_{1j}x_{1j}$, $\nu_{2}=\sum_{j=1}^{q}\omega_{2j}x_{2j}$ and $v_{3}=\sum_{j=1}^{k}\omega_{3j}y_{j}$, respectively; 3) in the inner estimation step, the endogenous latent variable *ѵ*_*η*_ were updated with *ν*_3_ = cov(*ν*_3_, *ν*_1_)*ν*_1_ + cov(*ν*_3_, *ν*_2_)*ν*_2_ + cov(*ν*_3_, *ν*_1_*ν*_2_)*ν*_1_*ν*_2_, furthermore the exogenous latent variables *ѵ*_1_ and *ѵ*_2_ by *ν*_1_ = cov(*ν*_1_, *ν*_3_)*ν*_3_ and *ν*_2_ = cov(*ν*_2_, *ν*_3_)*ν*_3_; 4) updating weights before moving to the next step: *w*_1*j*_ = *cov*(*x*_1*j*_, *ν*_1_), *w*_2*j*_ = *cov*(*x*_2*j*_, *ν*_2_) and *w*_3*j*_ = *cov*(*y*_*j*_, *ν*_3_). Steps 2)-4) were repeated until convergence (max (*w*_*ij* − *new*_ − *w*_*ij* − *old*_) < ∆, where ∆ is a convergence tolerance usually set at 0.0001 or less), and the outer weights were obtained. In addition, significant test of path coefficients and loadings were furnished by bootstrap procedures [24,25].

### Statistical significance

The modified statistics (mPLSPM) is defined as $U=\frac{\left|\gamma -0\right|}{\mathrm{se}\left(\gamma \right)}$, where *se* (*γ*) denotes the standard deviation of *γ*. Significance of parameter *γ* under the null hypothesis (*H*_0_): *γ* = 0 and the alternative hypothesis(*H*_1_): *γ* ≠ 0 is tested via a normal statistic in the form $U=\frac{\left|\gamma -0\right|}{\mathrm{se}\left(\gamma \right)}$, where se(*γ*) is calculated by the bootstrap procedures [29,30], since the distribution of parameters from modified PLSPM is unknown. The testing stages are as follows: 1) A large, pre-specified number of bootstrap samples (e.g. 1,000), each with the same number of subjects as the original sample, are generated via re-sampling with replacement. 2) Parameter estimation is done for each bootstrap sample using above modified algorithm, whose path coefficients or loadings can be viewed as drawings from their sampling distributions. All bootstrap samples together provided empirical estimators for the standard error of each parameter. 3) The result of bootstrapping procedure permits a *U*-test to be performed for the significance of the path coefficients or loadings, $U_{\mathrm{emp}}=\frac{\left|w-0\right|}{\mathrm{se}\left(w\right)}$ (for example $U_{\mathrm{int}\mathrm{er}}=\frac{\left|\gamma -0\right|}{\mathrm{se}\left(\gamma \right)}$ in Figure 1), where *U*_*emp*_ represents the empirical *U*-value, *w* (for example *γ* in Figure 1) denotes the original path coefficient or loading, and *se*(*w*) (for example *se*(*γ*) in Figure 1) indicates its bootstrapping standard error. The normal distribution provides the critical *U*-values at given α-levels. The histogram of the statistic was shown in Additional file 1: Figure S2.

### Simulation

**Figure 2:**
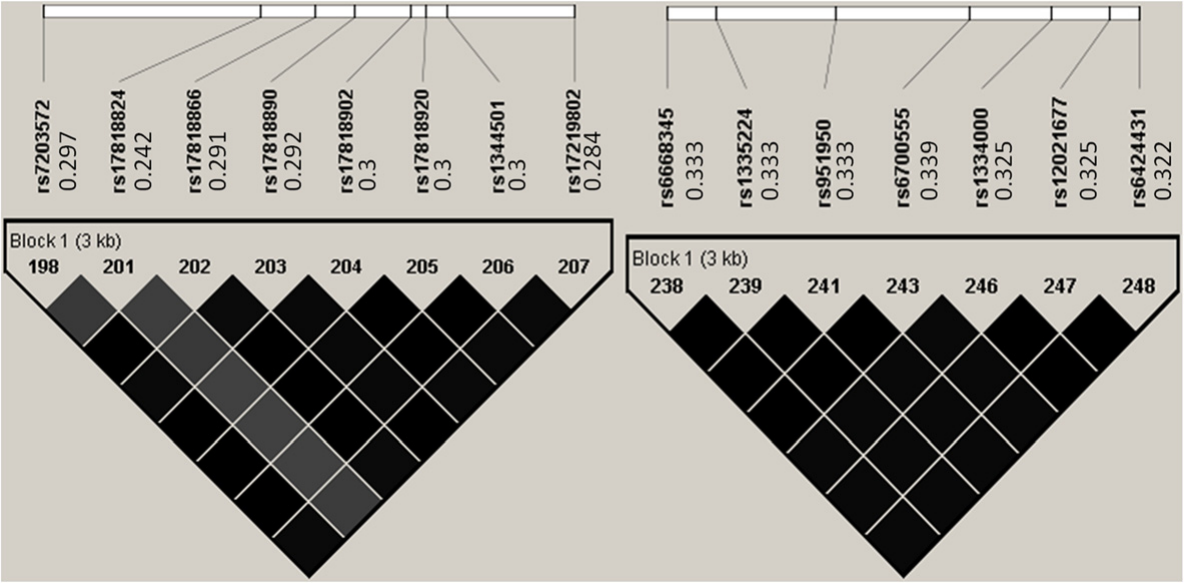
**Pair-wise r2 among the selected *FTO *region and *NEGR1*region.** The values to the right of the dbSNP IDs (rs# IDs) are the corresponding minor allele frequencies.

Simulation was conducted similar to a previous paper [31] as follows. Genotype data was generated by software gs2.0 [32] according to phase 1 and 2 *HapMap* data. Multiple phenotypic data were created to mirror the European Prospective Investigation of Cancer (EPIC)-Norfolk study [33,34] for which the waist circumference, hip circumference, and BMI were defined as multiple quantitative traits to reflect the body shape as the SLT. As noted earlier [31], the influence of body fat distribution has been linked with body shape named crudely after the fruits and vegetable(s) they resemble most (chilli, apple, pear, and pear apple) [35,36]. People with a larger waist have higher risks of hypertension, type 2 diabetes and high cholesterol than those who carry excess weight on the hips [10,11]. The combination of BMI, waist and hip circumferences is also a good predictor of cardiovascular risk and mortality [11,35,37]. In this paper, the simulated phenotype data was created based on abdominal obesity population from the EPIC-Norfolk study. The simulation procedure was detailed as follows:

(1) Phased haplotype data were downloaded from the *HapMap* web site (http://snp.cshl.org) on regions involved *FTO* (Chr16:52426867..52430604 with eight SNPs) and *NEGR1* (Chr1:71803870..71811085 with seven SNPs) on CEU population. Information on pair-wise *r*^*2*^ and minor allele frequencies is shown in Figure 2. Additive models were used for these SNPs. Based on the phased haplotypes, a large CEU population of 100,000 individuals was obtained via gs2.0 [32] with the 4th SNP of each region as the causal variants (called SNP1 and SNP2). In line with the current GWAS which are map-based rather than sequence-based, we removed the causal SNPs from simulated data to assess their indirect interaction effect on obesity related traits via correlated markers.

(2) As waist and waist to hip ratio (WHR) were commonly used to predict the type-II diabetes and cardiovascular disease [10,11,38,39], we created an abdominal obesity data set based on abdominal obesity sample (N = 355) in EPIC-Norfolk study. Multiple quantitative phenotypes with three traits (waist, hip, BMI) were generated from a trivariate normal distribution *Y* ~ *N*(*μ*, *Σ*) to assess our proposed statistic, where *Y* = (*y*_1_, *y*_2_, *y*_3_) was the random vector (waist, hip, BMI) for abdominal obesity types in EPIC-Norfolk study, with their sample mean $\bar{Y}$ = (105.2746, 106.0051, 29.2172) and covariance $\Sigma =\left(\begin{array}{l} 52.1991\;36.8688\;16.9545\\ 36.8688\;37.1419\;13.7969\\ 16.9545\;13.7969\;8.3859 \end{array}\right)$. The QQ-plots of the three variables (waist, hip, BMI) among the abdominal obesity groups are seen in Additional file 1. Supposed the causal SNPs’ interaction effect only on waist not on hip, under *H*_0_, the causal SNP1 and SNP2 had no interaction effect but main effect on BMI, thus $\mu =\left(\begin{array}{ccc} \text{wais}\hat{t}, & 106.0051, & 29.2172+0.32\times \mathrm{SNP}1+0.09\times \mathrm{SNP}2 \end{array}\right)$, where SNP1, SNP2 = 0, 1, 2 for three genotypes (GG, GA, and AA) at both loci, the main effect of SNP1 (0.32) and SNP2 (0.09) were assigned according to real data [40], and waist was estimated by an empirical model $\text{wais}\hat{t}=10.20345+0.62138*\text{hip}+0.99947*\mathrm{BMI}\;\left(F=568.25,P<0{.0001,R}^{2}=0.7635\right)$. Under *H*_1_, the interaction effect of two causal SNPs (SNP1 and SNP2) on BMI was *δ* kg/m^2^, thus ($\mu =\left(\begin{array}{ccc} \text{wais}\hat{t}, & 106.0051, & 29.2172+0.32\times \mathrm{SNP}1+0.09\times \mathrm{SNP}2+\delta \times \mathrm{SNP}1\times \mathrm{SNP}2 \end{array}\right)$. The range of the interaction effect *δ* = (0.10, 0.20, 0.30, 0.40, 0.50) was estimated by published data [41]. All simulation was performed by the R “***mvtnorm***” package available from CRAN (http://cran.r-project.org/).

(3) Under *H*_0_, 1,000 simulations given various sample sizes (*N* = 1000, 2000, 3000, 4000, 5000) were conducted to assess the type I error. Under *H*_1_, given *δ*, we repeated 1, 000 simulations under various sample sizes at two significant levels (*α* = 0.05, *α* = 0.01) to assess power of the mPLSPM statistic. The power of the proposed statistic for waist, WHR, and SLT was also estimated at given interaction effect *δ* under various sample sizes to compare their performance.

(4) To assess the performance of our proposed statistic, we compared it with a PCA-based linear regression model based on the ideas of three published work [20,26,28]. The PCA-based linear regression model was defined as $\eta =b+\sum_{i=1}^{P}\beta_{1i}U_{i}^{1}+\sum_{j=1}^{Q}\beta_{2j}U_{j}^{2}+\sum_{i=1}^{P}\sum_{j=1}^{Q}\gamma_{\mathrm{ij}}U_{i}^{1}U_{j}^{2}$ where *η* denoted the PCs of the three traits (waist, hip, and BMI), $U_{i}^{1},U_{j}^{2}$ represented the PCs for gene 1 and gene 2 respectively, and *P,Q* are the number of PCs in gene 1 and gene 2 chosen based on the proportion of variation explained. The pre-specified fraction of the total variance was 85% in this study.

### Application

Obesity is related to obstruction of food intake and energy balance regulation. The neurocenter in control of the food intake, hunger, and energy balance locates at hypothalamus and brainstem, and involves in a complicated neurochemical regulatory mechanism. The roles of both *TMEM18* gene and *BDNF* gene in the food intake and energy balance as with their association with obesity were shown [42-44]. Here we assess interaction of these two genes on obesity related quantitative traits. The genotype data of *TMEM18* (13 SNPs), *BDNF* (31 SNPs) and phenotype data (waist, hip, BMI) are from GWAS in the EPIC-Norfolk study (*N* = 2417). The EPIC-Norfolk study is a population-based, ethnically homogeneous, white Europe cohort study of 25,631 residents living in the city of Norwich, United Kingdom, and its surrounding area. Participants were 39–79 years old during the baseline health check between 1993 and 1997. Of these, 2417 individuals had complete genotype data for 2,500,000 SNPs on the whole genome [31,33]. The interaction between *TMEM18* and *BDNF* for waist, hip, BMI, WHR, body shape score 1 (BSS1, latent variable with waist, hip, and BMI as its manifest variables), and body shape score2 (BSS2, latent variable with BMI and WHR as its manifest variables) were detected using our proposed mPLSPM statistic at nominal level of *α* = 0.05.

## Results

### Simulation

#### Type I error rate

**Figure 3:**
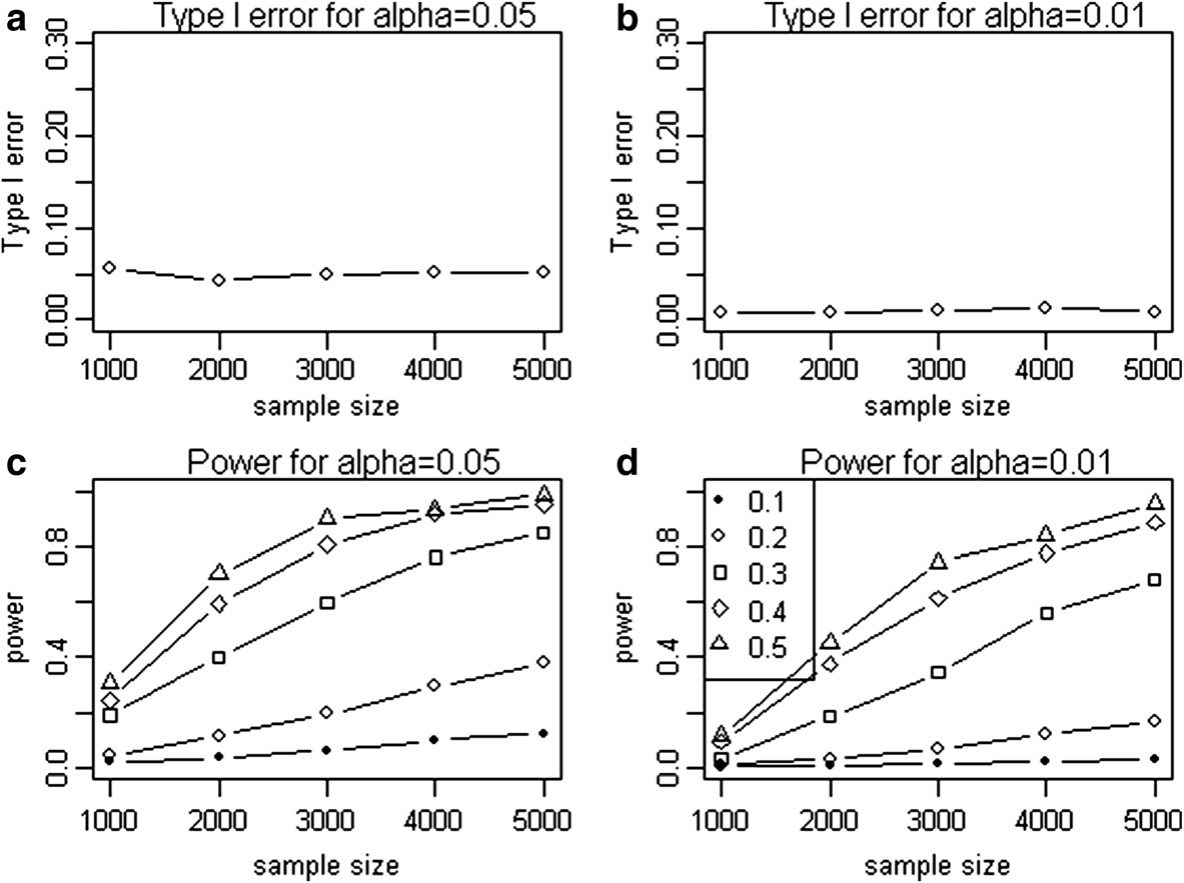
**Simulation results of type I error and power for the proposed mPLSPM statistic.** Type I error of mPLSPM statistic given different sample sizes under nominal level 0.05 **(a)** and 0.01 **(b)**; Power of mPLSPM statistic given different interaction effects and different sample sizes under nominal level 0.05 **(c)** and 0.01 **(d)**.

We first set out to verify the type I error rates of the mPLSPM statistic. In each simulation, a random sample of *N* individuals is drawn with *N* varying from 1000 to 5000 and consider two nominal significance levels, 0.01 and 0.05. For each parameter setting, we evaluate the type I error rate from 1,000 simulations. As shown in Figure 3a and 3b, type I errors of the mPLSPM statistic consistent with the nominal levels as a function of sample sizes.

#### Statistical power

To evaluate the statistical power of the mPLSPM statistic, we repeat simulations with various interaction effect *δ* and sample sizes. As expected, it monotonically increases with sample size and interaction effect (*δ*) under two given nominal levels (α = 0.05, α = 0.01) (Figure 3c and 3d).

**Figure 4:**
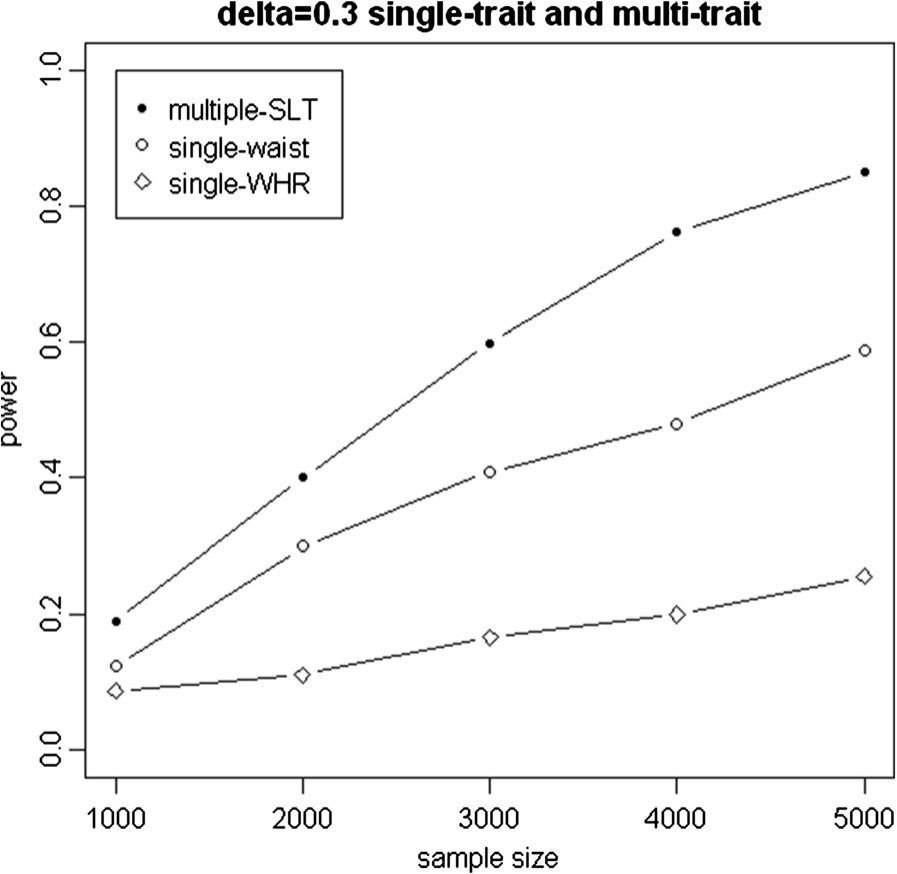
Power of mPLSPM statistic for body shape score, WHR, and waist.

Figure 4 shows power of the proposed statistic for waist, WHR, and SLT with given interaction effect *δ* =0.03 under various sample sizes. The power for body shape score is much higher than that for WHR or waist.

**Figure 5:**
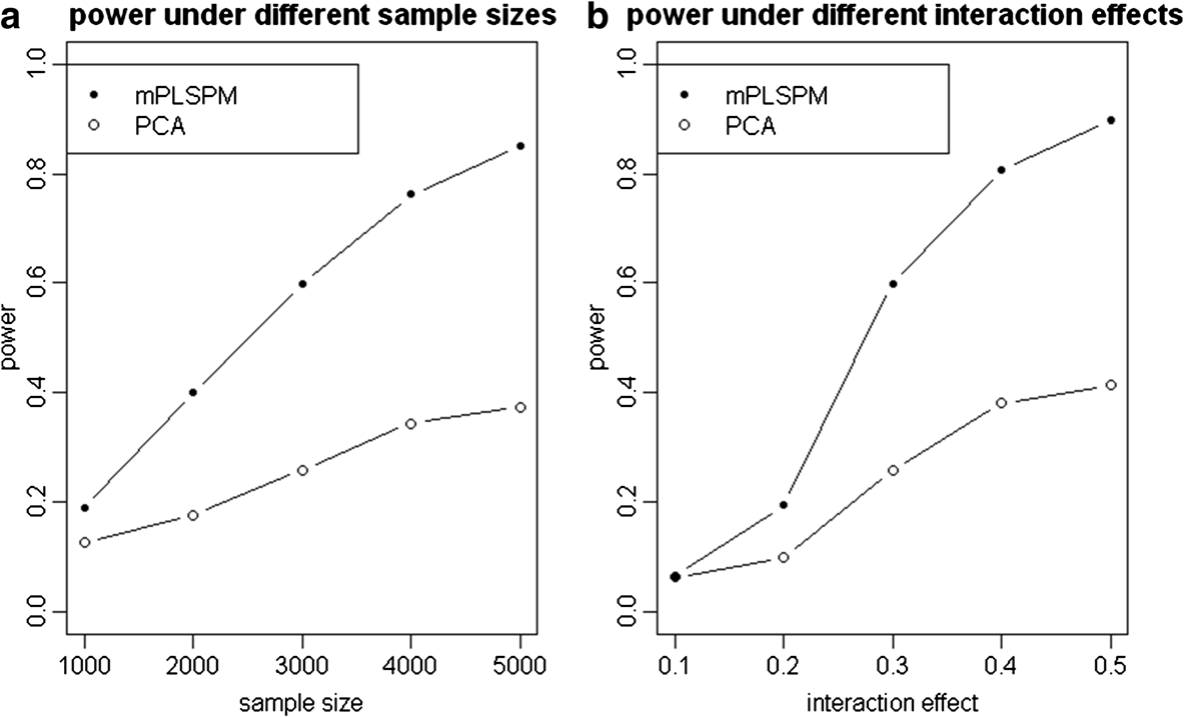
**Power of two methods under different sample sizes and different interaction effect. (a)** Power of mPLSPM statistic given an interaction effect of 0.3 and different sample sizes. **(b)** Power of mPLSPM statistic given different interaction effects and a sample size of 3,000.

**Figure 6:**
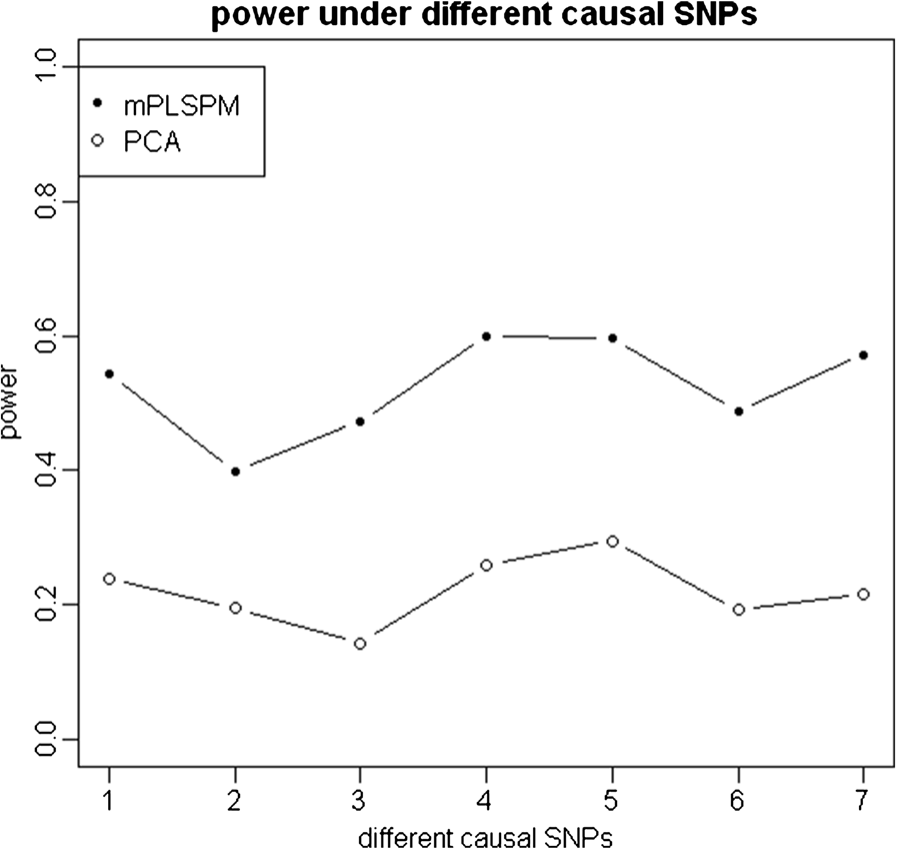
**Power of two methods under different causal SNPs.** Note: i (i = 1, 2, …, 7) denotes the causal SNPs are the i-th SNP in gene *FTO* and the i-th SNP in gene *NEGR1.*

Because of the first PCs of two genes explained a pre-specified fraction of the total variance (>85%), we use the first PC in the PCA-based test when comparing with the mPLSPM statistic. Figure 5 show the performances of the mPLSPM statistic and PCA-based linear regression as a function of different sample sizes and a fixed interaction effectand as a function of different interaction effect sizes and a given sample size of 3000 respectively. It can be seen that power increases monotonically with sample size and interaction effect size. Figure 6 gives their power given different causal SNPs with different minor allele frequencies and LD patterns, with the seven SNPs defined as the causal variant in turn. In all simulated scenarios, PCA-based test, which takes the approach of first collapsing markers in each of the two genes, is less powerful than the mPLSPM statistic (Figure 5, Figure 6), which may be due to a combination of the PCs not fully capturing the underlying interaction signals and the multiple degrees of freedom associated with that test statistic.

As one reviewer suggested additional simulations under the case that different SNPs affecting different phenotypes have also been conducted. Similar performance can be found (see Additional file 1: Table S2).

#### Application

**Figure 7:**
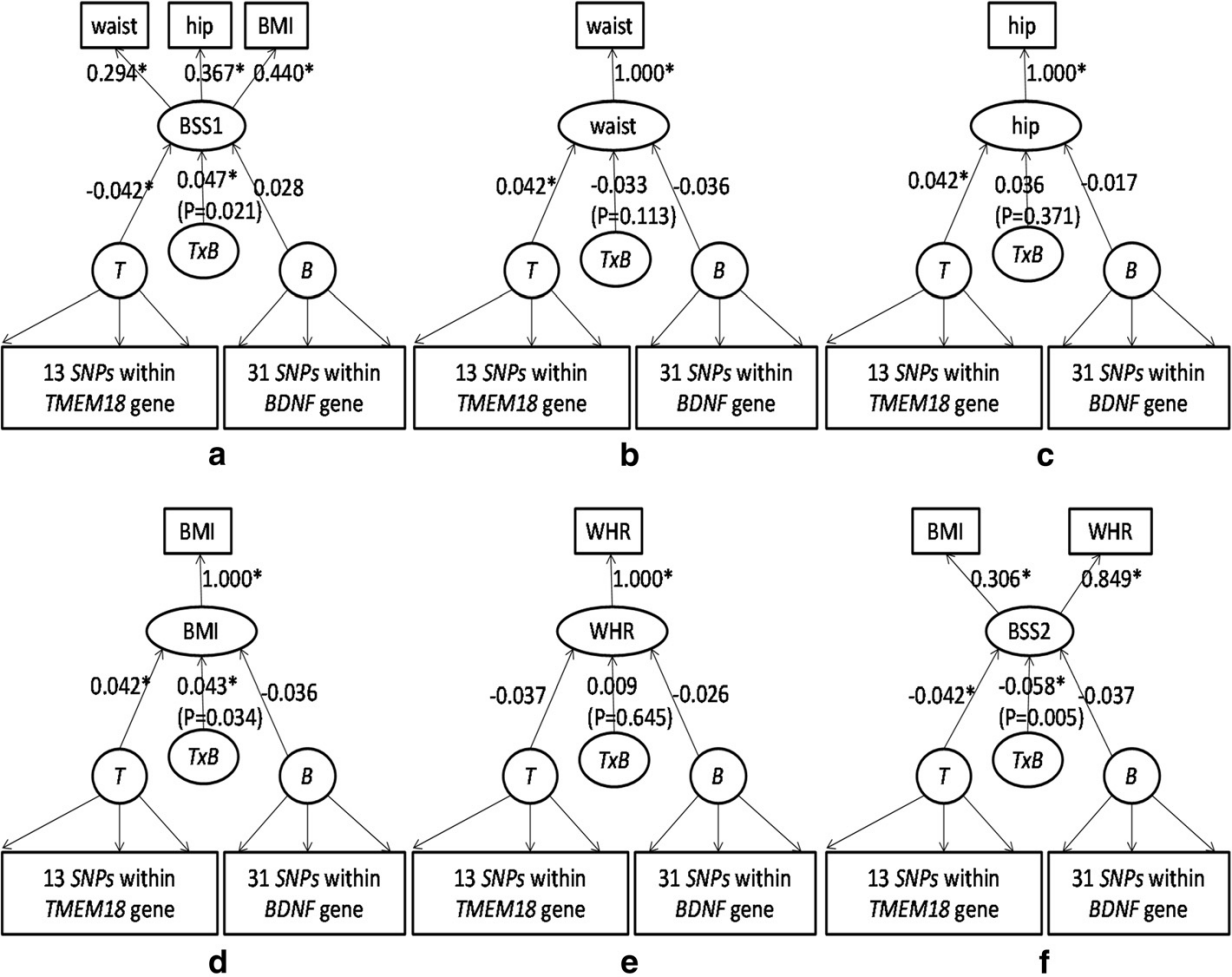
**Different models for*TMEM18*-*BDNF *interactions on obesity of the real data.** Different kinds of TMEM18-BDNF interactions on BSS1 **(a)**, waist **(b)**, hip **(c)**, BMI **(d)**, WHR **(e)**, and BSS2 **(f)**. Note:*P < 0.05.

We apply above two statistics to real quantitative traits data in the EPIC-Norfolk study. Different kinds of *TMEM18*-*BDNF* interactions on obesity using different modified PLSPM under standardization are shown in Figure 7. The interaction effect between the two genes on BSS1 (*γ* = 0.047), BSS2 (*γ* = 0.058) and BMI (*γ* = 0.043) are statistically significant with *P* = 0.021, *P* = 0.005, and *P* = 0.034 respectively (Figures 6d and 7a, 7f) though not for waist (*P* = 0.113), hip (*P* = 0.371), and WHR (*P* = 0.645) (Figure 7b, 7c, and 7e). Also available from Figure 7a, interaction between the two genes on single trait can be obtained as a product of the path coefficient (*γ*) and response loadings (λ), with 0.047 × 0.440 on BMI, 0.047 × 0.294 on waist, and 0.047 × 0.367 on hip, respectively.

PCA-based method has been also applied to detect different kinds of *TMEM18*-*BDNF* interactions on obesity. None showed statistical significance when using the first PC of each gene, while only interaction on BSS1 (*P* = 0.012) and BMI (*P* = 0.008) are statistically significant when using the first two PCs (explained over 85% of the total variance).

## Discussion

Under the hypothesis of thinking quantitatively [4], we have considered a general framework for gene-gene interaction on quantitative phenotype, which includes single SNP-SNP interaction on single trait, gene-gene (each with multiple SNPs) interaction on single trait and gene-gene (each with multiple SNPs) interaction on multiple traits, which was the most reasonable in genetic mechanism for multiple quantitative traits underlying complex diseases. In this paper, we furnished a novel mPLSPM statistic to detect the third of interaction. The mPLSPM statistic should alleviate the burden of single SNP- single trait paradigm which inevitably has high false positive rate due to multiplicity problem, as well as its reduction of power due to the underuse of the LD information [16,17]. Furthermore, the new approach does not have the drawback of gene (multiple SNPs)-single trait paradigm for reasons mentioned earlier, and for most complex diseases (type II diabetes, obesity, disturbance of consciousness), although their quantitative phenotype could in principle be measured, they might not be used for practical reasons (quantitative phenotypes are “really there” but hidden). Our proposed statistic uses the framework of SLT as a quantitative phenotype which was inferred from observed variables (multiple SNPs within gene regions, and multiple traits of a specific complex disease). Through simulation it was shown that the proposed novel mPLSPM statistic to be not only powerful (Figure 3c, 3d) but superior to the PCA-based linear regression method (Figure 5a, 5b, 6).

After applying the novel statistic to the real data, a significant *TMEM18*-*BDNF* interaction has been shown for body shape score as a SLT but not for its individual components (waist, hip, and WHR) (Figure 7a-7f), suggesting that the SLT (body shape score) to be more suitable to capture the interaction effect than any single trait. The biological significance in the food intake and energy balance regulation system is in line with the literature, and these two genes have been confirmed to be associated with obesity [42-44].

Our approach shares similarity with traditional SEM, available as either covariance-based or component-based [25,45,46]. However, gene-based multiple SNPs with high LD in genomic data and multiple high correlated traits, the covariance-based SEM suffers from the strong multicollinearity between them. Our use of PLSPM is a component-based with the following advantages: 1) use of reflective measurement model to avoid the impact of high multicollinearity among multiple SNPs, and among multiple traits; 2) as a “soft modeling” approach (very few distribution assumptions, variables can be numerical, ordinal or nominal, and no need for normality assumptions) suitable for any genetic model (additive, recessive, dominant, etc.) [23,24,47]. However, the usual PLSPM cannot handle the interaction between latent variables straightforwardly, the modified PLSPM has a product term of combined multiple SNPs effect within two genes (gene A and gene B).

A reviewer has also indicated that another way to test interaction would be to add a new latent variable for all the pair-wised SNP × SNP interactions to the path modeling and test whether the path coefficient from this interaction latent variable to the latent trait variable is significant [48]. We compared this method with our proposed statistic, and results showed they have similar performance (see Additional file1: Table S1). However, when the number of SNPs is large, there will be so many SNP × SNP terms and undoubtedly bringing us higher computation burden. Our method seems more practical in real data analysis. It is worth mentioning that our proposed method should only be used for testing the interaction, but not for detecting main effect. Testing multiple-traits may only be superior if pleiotropic SNPs and genetic related traits exist, and when the number of traits is large or the correlation (or LD) structure among the traits is small, the power of our statistic will decrease.

A possible drawback of the proposed approach is the computing time spending on bootstrap test used to evaluate the standard deviation of path coefficients. Ideally, a parametric statistic can be developed in the near future. Our findings on the interaction also call for replications by other studies.

## Conclusions

The proposed novel mPLSPM statistic is a valid and powerful gene-based method for detecting gene-gene interaction on multiple quantitative phenotypes. Further work is needed to make its use in GWAS more practical.

## Abbreviations

mPLSPM: modified Partial Least Squares Path Modeling; SLT: Synthetically latent trait.

## Competing interests

The authors declare that they have no competing interests.

## Authors’ contributions

FYL, JHZ, ZSY, XSZ, JDJ and FZX conceptualized the study, acquired and analyzed the data and prepared for the manuscript. All authors approved the final manuscript.

## Supplementary Material

Additional file 1 **Introduction for Partial least squares path model and some additional results.** The first part is an introduction for PLSPM, Additional file 1: Figures S2-S5 and Tables S1-S2 are some additional results which the reviewers indicated us to add. Click here for file
