# Supplementary material for: A powerful latent variable method for detecting and characterizing gene-based gene-gene interaction on multiple quantitative traits
Source: BMC Genet. 2013 Sep 23;14:89. doi: 10.1186/1471-2156-14-89 (PMC3848962; doi:10.1186/1471-2156-14-89)
Supplement: Additional file 1 — Introduction for Partial least squares path model and some additional results. The first part is an introduction for PLSPM, Additional file 1: Figures S2-S5 and Tables S1-S2 are some additional results which the reviewers indicated us to add. [file 1471-2156-14-89-S1.docx]

**Supplementary materials**

**Introduction for Partial least squares path model (PLSPM)**

**Model framework -- *GWAS for body shape as an example***

This model framework is based on PLSPM which developed from structural equation models (SEM). SEM are complex models allowing the study of real world complexity by taking into account a whole number of causal relationships among latent concepts (i.e. the latent variables (LVs)), each measured by several observed indicators usually defined as manifest variables (MVs).

Currently, two complementary schools come to the fore in the field of SEM: covariance-based SEM and component-based SEM. Covariance-based SEM can be considered as a generalization of path models, principal component analysis (PCA) and factor analysis to the case of several data tables connected by causal links. It is usually used with an objective of model validation. Component-based SEM is a partial information method with two-steps: 1) latent variables (LV) scores are computed using Partial Least Squares (PLS) algorithm and 2) ordinary least squares regressions (OLS) are carried out on LV scores for estimating the structural equations. It can be considered as a generalization of PCA to the case of several data tables connected by causal links. It is mainly used for score computation (such as *body shape score (BSS)* in this study).

Each path-modeling-based statistic in Figure S1 is formed by 2 sub-models: Structural (Inner) model and measurement (Outer) model. The structural model indicates the relationships among the latent variables ($\xi_{1}$,$\xi_{2}$and$\xi_{3}$), and in this study we add a product term $\xi_{1}\times\xi_{2}$to detect interaction, both of them are inferred from the observed SNPs (from gene A and gene B) and traits (waist, hip, BMI) respectively. The association between two genes ($\xi_{1}$,$\xi_{2}$, and $\xi_{1}\times\xi_{2}$) and body shape ($\xi_{3}$) are measured by the path coefficient ($\beta_{31}$,$\beta_{32}$,and $\gamma$). The measurement model formulation depends on the direction of the relationships between the latent variables ($\xi_{1}$,$\xi_{2}$,or$\xi_{3}$) and the corresponding manifest variables(SNP11,…, SNP1p, SNP21,…,SNP2q,waist,hip,BMI).The gene A ($\xi_{1}$) and gene B ($\xi_{2}$) are defined by aggregating the small effects of $p$SNPs(SNP11,…,SNP1p) and $q$SNPs (SNP21,…,SNP2q), while the BSS ($\xi_{3}$) are defined by traits (waist, hip, BMI). The effect of a specific SNP on its relevant gene ($\xi_{1}$,$\xi_{2}$) can be determined by the loading vectors ($\lambda_{11},\lambda_{21},...,\lambda_{p1}$) and ($\lambda_{12},\lambda_{22},...,\lambda_{q2}$). Similarly, the *BSS* ($\xi_{3}$) related to body shape trait can be determined by the loading vector ($\lambda_{13},\lambda_{23},\lambda_{33}$).

As a matter of fact, different types of measurement model are available: the *reflective model* (or outwards directed model), the *formative model* (or inwards directed model) and the *MIMIC model* (a mixture of the two previous models).The *reflective model* has causal relationships from the latent variable to the manifest variables in its block. Thus, each manifest variable in a certain measurement model is assumed to be generated as a linear function of its latent variables and residual ($\varepsilon$). As a matter of fact, since latent and manifest variables are standardized, the location parameters in the mPLSPM statistic can be discarded in OLS simple regressions. Thus, it is not affected by multicollinearity. For example, each manifest variable (waist, hip, BMI) for body shape ($\xi_{3}$) can be denoted as:$waist=\lambda_{13}\cdot\xi_{3}+\varepsilon_{13}$,$hip=\lambda_{23}\cdot\xi_{3}+\varepsilon_{23}$, and$BMI=\lambda_{33}\cdot\xi_{3}+\varepsilon_{33}$. In addition, construction of *reflective* model give rise to observed (manifest) variables with unidimensional form based on factor analysis model and aims at accounting for observed variances or co-variances, therefore the MVs reflect (effect on) the LV. In contrast to *reflective* (or effects) model, the *formative* (causal) model has causal relationships from the manifest variables to the latent variables, namely the LV is caused (formed) by the MVs. Its construction is combination of observed (manifest) variables with multidimensional form and aims at minimizing residuals in structural relationships to explain the unobserved (latent) variable with higher $R^{2}$.The location parameters in formative model cannot be discarded in OLS multiple regressions, so it is affected by multicollinearity. For example, if the mPLSPM statistic in Figure S1 were *formative*, then body shape ($\xi_{3}$) can be denoted as:$\xi_{3}=\lambda_{31}\cdot waist+\lambda_{32}\cdot hip+\lambda_{33}\cdot BMI+\varepsilon_{3}.$ In the case of high multicollinearity, the parameters in formative model are estimated by PLS regression. Since the aim of mPLSPM statistic is mainly to capture the association between effect of SNPs set (genome region) and effect of traits (body shape), and after using “*Cronbach’s alpha*” tool for checking, the blocks meet homogeneity and unidimensionality. So we use the *reflective* model to set up the measurement model.

Basically, three parameters are needed to estimate in the PLSPM statistic model: **1)** Estimate the LV scores ($\xi$) using linear combinations of their MVs, obtained by an iterative algorithm based on simple / multiple least squares regressions. **2)** Estimate the path coefficients ($\beta$’s, and$\gamma$) using regression between dependent LV ($\xi_{3}$) and independent LVs (including$\xi_{1}$, $\xi_{2}$and their product term$\xi_{1}\times\xi_{2}$) obtained by least squares regression or PLS regressions (with higher multicollinearity between independent LVs). **3)** Estimate the loadings ($\lambda$’s) using regressions of each block of MVs with its LV, obtained by least squares regressions.

**Statistical interpretation for the parameter in the mPLSPM model**

All the path coefficients and loadings within the PLSPM model are standardized; therefore, their effects can be compared with each other. Interpretation can be performed by the modeling structure (Figure S1): 1) the main effects of two genes ($\xi_{1}$,$\xi_{2}$) on body shape ($\xi_{3}$) can be measured by the path coefficient ($\beta_{31}$,$\beta_{32}$) in the structure (inner) model, similarly $\gamma$for the interaction effect. 2) The $R^{2}$ of the body shape ($\xi_{3}$) measures the variance proportion interpreted by its exogenous latent variables ($\xi_{1}$,$\xi_{2}$) and their product term$\xi_{1}\times\xi_{2}$. 3) The interaction effect of the two genes on a single trait can also measured by the product of loadings and coefficients along the path (for example, the interaction effect of two genes on BMI is ($\gamma\cdot\lambda_{33}$). 4) The SLT of body shape was measured by *BSS* ($\xi_{3}$), which is the combination of waist, hip and BMI with their weights as$\lambda_{13}$,$\lambda_{23}$, and $\lambda_{33}$ respectively. Furthermore, as the measurement (outer) model of body shape are *reflective* and the loadings are standardized, the relationship between traits and body shape can be denoted by a simple linear function:

$wais\hat{t}=wais\bar{t}+\lambda_{13}s_{waist}\cdot\xi_{3}$, $hi\hat{p}=hi\bar{p}+\lambda_{23}s_{hip}\cdot\xi_{3}$, $BM\hat{I}=BM\bar{I}+\lambda_{33}s_{BMI}\cdot\xi_{3}$.

Reference:

Esposito VV, Chin WW, Henseler J, Wang H (2010) Handbook of Partial Least

Squares: Concepts, Methdos and Applications. *Berlin Heidelberg: Springer*.


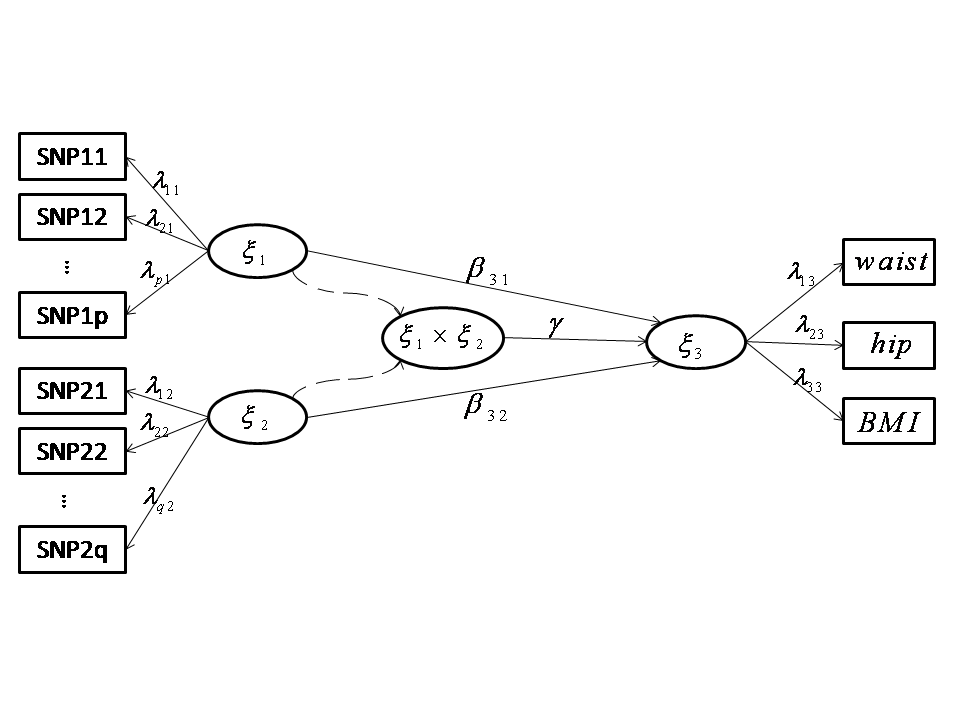


Figure S1 The framework of PLSPM for body shape as an example.


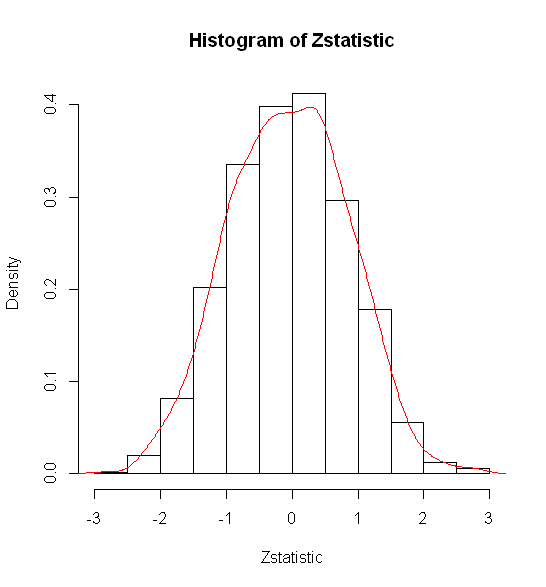


Figure S2 The histogram of the test statistics using bootstrap.


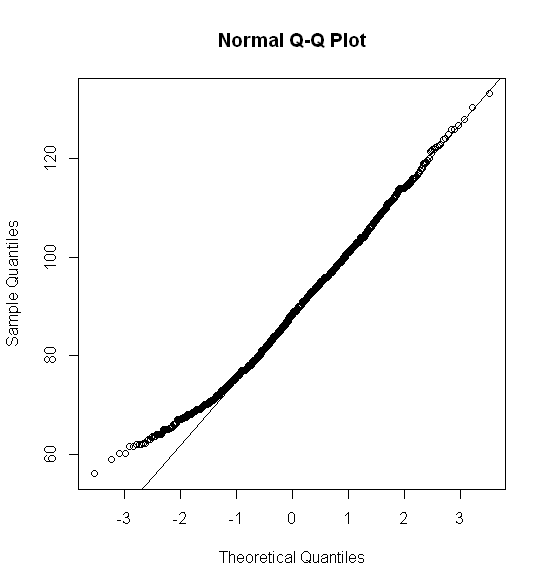


Figure S3 The QQ-plots of the waist variable among the abdominal obesity groups.


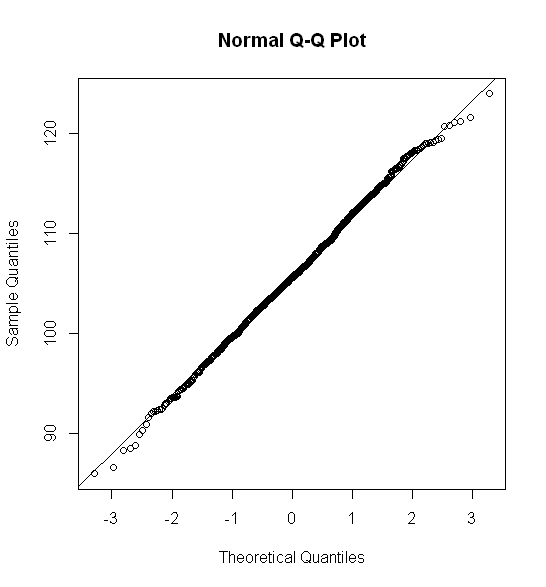


Figure S4 The QQ-plots of the hip variable among the abdominal obesity groups.


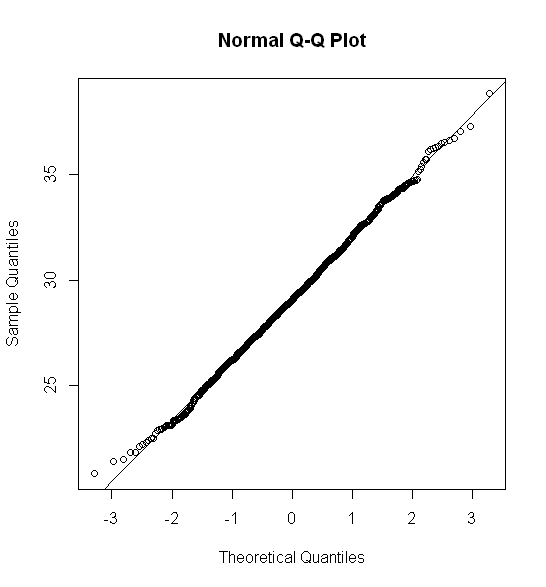


Figure S5 The QQ-plots of the BMI variable among the abdominal obesity groups.

Table S1 Power of two interaction methods

| Sample size | 1000 | 2000 | 3000 | 4000 | 5000 |
| --- | --- | --- | --- | --- | --- |
| mPLSPM | 0.190 | 0.400 | 0.599 | 0.762 | 0.851 |
| SNP×SNP | 0.192 | 0.391 | 0.612 | 0.755 | 0.863 |

Table S2 Power of two different cases

|  | BSS | waist | hip | BMI | WHR |
| --- | --- | --- | --- | --- | --- |
| Case1 | 0.599 | 0.166 | 0.042 | 0.705 | 0.409 |
| Case2 | 0.463 | 0.421 | 0.036 | 0.468 | 0.328 |

Note: Under case1, we selected same casual SNPs for waist and BMI. Under case2 we used different causal SNPs for them

(waist =105.2746+0.28*SNP_1_2+0.3*SNP_2_5+0.5* SNP_1_2*SNP_2_5 and

BMI=29.2172+0.28*SNP_1_4+0.3*SNP_2_4+0.3* SNP_1_4*SNP_2_4, SNP_i_j : the j-th SNP in gene i)*.*
